# Effectiveness of Roflumilast in Treating Chronic Obstructive Pulmonary Disease: A Systematic Review and Meta-Analysis

**DOI:** 10.7759/cureus.22843

**Published:** 2022-03-04

**Authors:** Sumbal Naseem, Muhammad Hassan, Shazia Nasim Akhtar, Fibhaa Syed, Naveed Ullah Khan, Muhammad Usman

**Affiliations:** 1 Respiratory Medicine, University of Chester, Chester, GBR; 2 Neurology, Shaheed Zulfiqar Ali Bhutto Medical University, Islamabad, PAK; 3 Intensive Care Unit, King's College Hospital NHS Foundation Trust, London, GBR; 4 Internal Medicine, Shaheed Zulfiqar Ali Bhutto Medical University, Islamabad, PAK

**Keywords:** systematic review and meta analysis, safety, efficacy, chronic obstructive pulmonary disease, roflumilast

## Abstract

Background

Chronic obstructive pulmonary disease (COPD) is a chronic airflow obstructive condition. The mainstay of treatment is to avoid exacerbation and manage the symptoms. Roflumilast is being used as a part of treatment to reduce the inflammatory process in this disease.

Method

This systematic review and meta-analysis were conducted following the provided guidelines. PubMed, Cochrane Library, and Cinahl were considered for searching the desired studies selected until 19 June 2021. The eligibility criteria for inclusion and exclusion were set before selecting studies.

Result

Five hundred eighty (580) studies were identified at the beginning. Removal of duplicates was done using Endnote software. The eligibility criteria, including the randomized controlled trial study design and others, were applied for screening the title and abstracts. Six studies were selected for the qualitative analysis. After assessing the data from these studies, it was found that roflumilast is an effective drug to treat COPD. Roflumilast plays an essential role in improving quality of life, inflammatory process, and clinical improvement. The drug's mild to moderate adverse effects were observed, but no significant severe adverse events were reported, and the drug was well tolerated.

Conclusion

Roflumilast is a valuable drug that can be used for its beneficial effects on COPD exacerbation. The benefits of the drug outweigh its adverse effects.

## Introduction and background

Chronic obstructive pulmonary disease (COPD) is a chronic airflow obstructive condition with a progressive inflammation of the airways [1]. It is an economic burden worldwide. COPD affects one in seven people in the UK, predominantly in the age group above 50 years [2]. COPD- related death rates are higher in men than in women. COPD is a preventable disease; approximately around 50% of the cases are attributable to smoking and the remaining 50% to other factors [3]. The mainstay of treatment of COPD depends on treating the symptoms and slowing the progression of the disease [4].

Roflumilast is an orally administered selective phosphodiesterase-4 inhibitor (PDE4 inhibitor). It increases intracellular 3′,5′-cyclic adenosine monophosphate levels in inflammatory cells and the epithelial cells of the airways, which may contribute to the reduction of pulmonary inflammation [4]. It is a potent drug that can be used once daily. Several studies have shown that roflumilast in combination with long acting beta 2 agonists and long acting muscarinic antagonists could be helpful in treating COPD [5]. Another study stated that roflumilast helps avoid COPD exacerbation and improves the quality of life [6].

This study was conducted to establish the effectiveness of roflumilast in COPD patients, researchers, and clinicians to regularize its use in its management. This study focuses on the recent studies highlighting the efficacy of roflumilast and includes quantitative analysis.

## Review

Search strategy

Three different databases were utilized to cover the literature: PubMed, Cinahl, and Cochrane Library. The studies were taken from 2001 to 2021. For searching, the words and their synonyms used in PubMed were: ((roflumilast or daliresp) AND (effect or outcome)) AND (COPD or "Chronic obstructive lung disease" or "Chronic obstructive pulmonary disease"). In Cochrane ((roflumilast or daliresp) AND (effect or safety or outcome)) AND (COPD or "Chronic obstructive lung disease" or "Chronic obstructive pulmonary disease") title abstract were key words. In Cinahl key search was: ((roflumilast or daliresp) AND (effect or safety or outcome)) AND (COPD or "Chronic obstructive lung disease" or "Chronic obstructive pulmonary disease"). The filter applied was only English language, and other exclusion criteria were applied later. Grey literature is not included in this study. No author was contacted to get access to the full text.

Inclusion and exclusion criteria

The screening was followed by prior settled inclusion and exclusion criteria. It was done by only one reviewer on 19 June 2021. The studies eligible for this systematic review are randomized control trials. The subjects included were COPD patients (smoker/non-smokers), smokers, or ex-smokers. In some included studies, COPD was presented with chronic bronchitis. Some studies were also included in which roflumilast was compared to placebo and in few studies different doses of roflumilast were compared to observe its effect on COPD. The exclusion criteria included all other study designs like case reports, editorials and posters, studies with irrelevant or incomplete results, studies with non-human subjects like animals or cell cultures, duplicate studies, and those studies in which English was not the medium, e.g., French and Chinese (Table 1).

**Table 1:** Inclusion and exclusion eligibility criteria for this systematic review

INCLUSION CRITERIA	EXCLUSION CRITERIA
Randomized controlled trials	Case reports reviews and editorials
Only adult human subjects	Non-human subjects
Only English studies	Non-English studies
Studies that compared roflumilast with placebo or with different doses of roflumilast	Duplicate studies or Irrelevant or incomplete outcomes

Outcome measurements

The outcomes for this systematic review were the quality of life and clinical improvement, measured and observed by spirometry at the time of follow-ups and the status of inflammatory markers seen in blood and sputum by comparing the statistical significance of roflumilast and placebo. Adverse events were reported for tolerability and safety outcomes (Table 2).

**Table 2:** Characteristics and jaded scoring of the included studies Abbreviations: RCT= randomized control trial, DB= double blind, ↓= decreased, URTI= upper respiratory tract infection, COPD= chronic obstructive pulmonary disease, OD= once daily, EOD= every other day, µg= microgram Outcomes: quality of life= 1, clinical improvement=2, inflammatory cell load=3

Author	Origin of study	Study design	Study size	Participant’s characteristics	Treatment group	Control group	Follow-up	outcome	Adverse effects	Jaded score
Grootendorst et al [7]	Netherlands	RCT DB Crossover	38	COPD Smoker/ ex- smokers	Roflumilast 500µg OD	Placebo 500µg OD	Once weekly	2,3	Diarrhea, nausea, dyspepsia, vomiting, headache, dizziness, cough, dyspnoea, URTI, chest pain, palpitation abnormal ECG, thrombophlebitis	4
Liu et al [8]	China	RCT DB two-arm	120	COPD	Roflumilast 500µg OD	Placebo 500µg OD	After 12 months and then after 3 months	1,2	URTI, Diarrhoea, ↓ weight, anorexia, COPD exacerbation, gastritis, constipation, rhinnorhea, dizziness	4
Wells et al [9]	UK	RCT	27	COPD with Chronic bronchitis	Roflumilast 500 µg OD	Placebo 500µg OD	0,1,4,8,12,14 weeks	1,3	Nausea, diarrhoea, ↓weight, URTI, cough, pleurisy, pneumonia, insomnia	3
Lee et al [10]	Korea	RCT DB phase III trial	207	COPD Smoker/ex- smokers	Roflumilast 500 µg OD	Placebo 500µg OD	0,4,8,12 weeks	2	URTI, Diarrhoea, ↓ weight, anorexia, COPD exacerbation, Gastritis, constipation, rhinorrhea, dizziness	2
Mackay et al [11]	UK	RCT DB phase II	81	COPD with Chronic bronchitis, Smoker	Roflumilast 500 µg OD prednisolone 30mg OD for 10 days and amoxicillin 500mg TDS for 7 days	Placebo 500µg OD & prednisolone 30mg OD for 10 days & amoxicillin 500mg TDS for 7 days	Day 1,7,14,28,56	2,3	COPD exacerbation Diarrhoea, insomnia, ↓weight	2
Watz et al [12]	Multi centers in 15 countries	RCT DB	1321	COPD, Smoker	Arm 1 Roflumilast 500 µg OD	Arm 2: Roflumilast 250µg OD 4 weeks then 500 µg Arm 3: Roflumilast 500 µg EOD then OD	2,4,8 weeks	2,3	Diarrhoea, nausea, headache, ↓appetite,insomnia abdominal pain	4

Reviewing process

The Preferred Reporting Items for Systematic Reviews and Meta-Analyses (PRISMA) flowchart was followed for the reviewing process. All the searched data for studies was mined from three databases, i.e., PubMed, Cochrane Library, and Cinahl, into the software Endnote program, and duplicates were removed. Only one reviewer screened titles and abstracts for the required study design, i.e., randomized controlled trial keeping in view the eligibility criteria for inclusion and exclusion. Only accessible databases and studies are reviewed in this study. The PRISMA template was used throughout the review to follow eligibility criteria.

Data extraction

All the data from the selected studies were extracted into the Excel program on spreadsheets. The information taken from the studies were study name and origin, subject’s characteristics, study design, doses, follow-ups, and study outcomes (Table 2).

Study bias

The assessment of the bias and risk was done in all selected studies using the Jadad scoring tool. The author applied the Jadad score for calculating the risk of bias which has components: randomization, appropriate randomization, blinding, proper blinding, and the score for dealing with missing information.

Results

**Figure 1:**
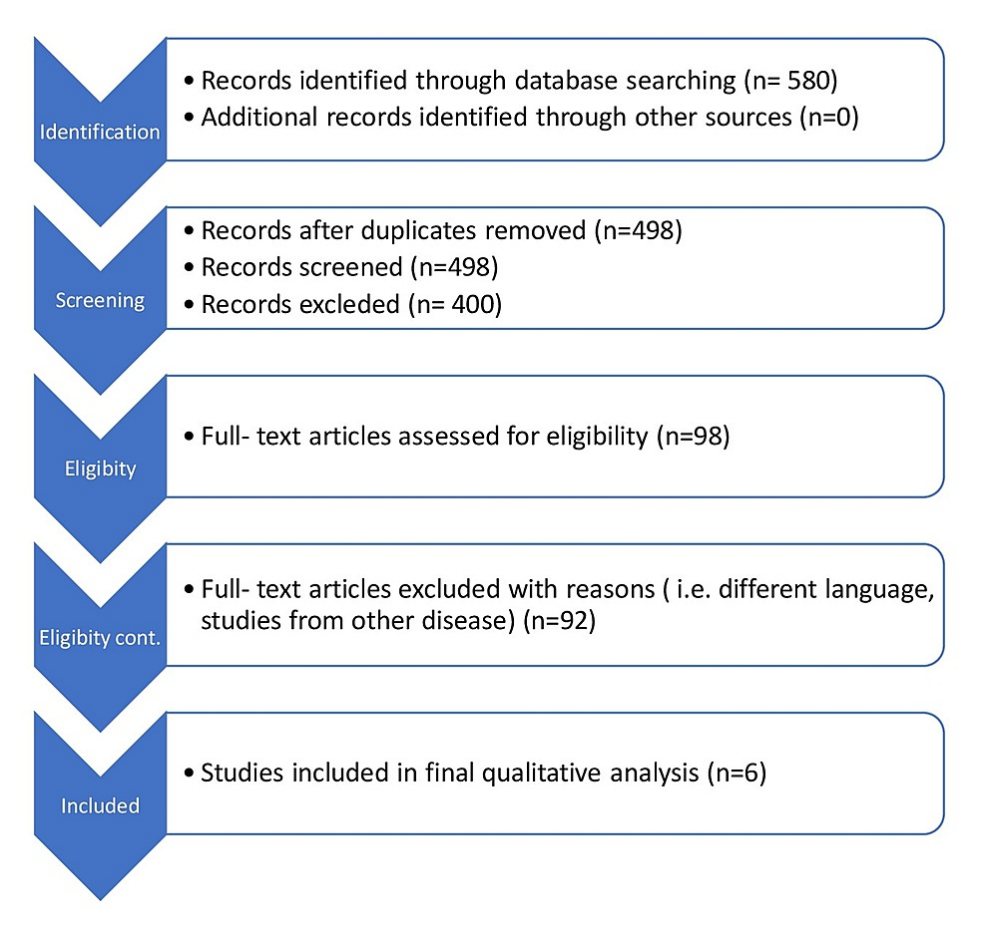
PRISMA flowchart for identifying the selected studies PRISMA: Preferred Reporting Items for Systematic Reviews and Meta-Analyses

After searching through three different accessible databases using keywords and their synonyms, 580 studies were found. No other databases were used, and no author was contacted for the required study. After duplicated studies remaining 498 were scanned according to eligibility criteria of inclusion and exclusion. After applying the criteria, only complete text studies were screened, and at the end, six studies were selected for this systematic review (Figure 1). The methodology of selected studies was screened for outcomes by only one author.

All the studies in this review are randomized controlled trials taken from Asia and Europe. Two studies were done internationally, Wells et al. [9] took place in the UK, Lee et al. [10] was from a Korean cohort, and Mackay et al. [11] also took place in UK. Grootendorst et al. [8] and Liu et al. [9] originated in Netherland and China. In contrast, Watz et al. [12] was done in multi-centers across 15 countries. All studies were scored according to the Jadad scoring system. Four studies [1-3,6] used proper blinding with appropriate randomization, while only one study [3] used proper blinding without appropriate randomization. The dose of roflumilast is similar in five out of six studies. The legal regime was given in Mackay et al. [11] with roflumilast. Watz et al. [12] used different doses of roflumilast for the three arms. The sample size was quite variable for all selected studies ranging from minimal 27 to 1321 (Table 2).

Quality of life

The improvement in quality of life in patients with COPD after administration of roflumilast was compared with placebo of the exact dosage in two studies. Liu et al. [9] measured by St. George’s Respiratory Questionnaire (SGRQ) scale, which showed significant improvement at the follow-ups as compared to the placebo group. Wells et al. [10] used SGRQ and included forced expiratory volume (FEV1), cough, dyspnea, 6-minute-walk distance (6MWD), and breath, cough, and sputum scale (BCSS) questionnaires, to compare roflumilast with the placebo group, although it showed improvement but not to a significant level.

Clinical improvement

The clinical improvement was measured in five out of six studies [8,9,11-13]. The patients with COPD were administered roflumilast and compared to placebo or comparison was between different dosage groups of roflumilast. Grootendorst et al. [7] included patients with COPD who were smoking presently, and some were ex-smokers. In Wells et al. [9], participants were COPD with chronic bronchitis, while Liu et al. [8] included only COPD patients. All three studies showed significant improvement in the pulmonary function tests i.e. FEV1/forced vital capacity (FVC) ratio. Patients in Mackay et al. [11] and Watz et al. [12] taking roflumilast showed improved pulmonary function test compared to placebo, but it was not significantly improved.

Inflammatory cell load

Grootendorst et al. [7], Watz et al. [12], and Mackay et al. [11] showed the response of inflammatory markers present in sputum and blood while administrating roflumilast and placebo to COPD patients. Grootendorst et al. [7] showed significant improvement in patients taking roflumilast blood markers (neutrophils and eosinophils); Wells et al. [9] illustrated that significant improvement was seen in sputum markers (neutrophils in sputum) at the end of the study as compared to placebo. In comparison, Mackay et al. [11] showed no significant difference in both the roflumilast and placebo groups.

Adverse effects

The tolerability and adverse events were mentioned and calculated by all authors for the selected six studies. The mentioned adverse effects were mild to moderate, and no significant adverse effect was seen; five studies compared roflumilast with placebo while Watz et al. [12] compared three groups of different doses of roflumilast, illustrating that gradually escalating the dose can reduce the side effects.

Discussion

In this review, six studies were assessed for the effectiveness of roflumilast in COPD patients. It was done by evaluating the effect on the patients by assessing the quality of life, clinical improvement, adverse events hindering the treatment, and the effect of roflumilast on inflammatory markers.

The quality of life was improved after administrating roflumilast in the subjects with COPD was assessed in two studies (Table 2). In both studies, Liu et al. [9] and Wells et al. [9], the SGRQ questionnaire [13] was used. Both studies showed improvement in quality of life, but the p-value in Wells et al. [9] was more significant than 0.05.

FEV1 is the gold standard for diagnosing and staging COPD and has been used as a primary measurement of lung function in clinical trials [14]. Although a minimum clinically important difference (MCID) for FEV1 is not yet established, the suggested MCID for FEV1 is 100-140 mL in patients with COPD [15]. In this study, the clinical improvement was analyzed using FEV1 after starting the administration of roflumilast. In five out of six studies [8,9,11-13] except Wells et al. [9], all the studies showed significant clinical improvement. Liu et al. [9] and Lee et al. [10] showed p < 0.01, while Grootendorst et al. [7] roflumilast showed a p-value of 0.0001, and the placebo had p = 0.018. Mackay et al. [11] observed a statistically significant value of 0.005, whereas Watz et al. [12] stated that clinically patients were improved in its three arms of grouping no matter what dose was given for what duration. Thus, it is explained in all studies reviewed that roflumilast produce good clinical outcomes. However, Lee et al. [10] and Mackay et al. [11] have a Jadad score of 2, so their reliability and risk of bias can be doubted. If the study uses blinding and proper blinding, it sources the evidence for reliability.

Roflumilast is a PDE4 inhibitor that decreases inflammation of the airway [16]. In this study, the effect on inflammatory markers was discussed by three studies as an outcome. Mackay et al. [11] found that there was no significant effect on neutrophil counts in sputum after administration of roflumilast. Wells et al. [9] stated that although neutrophil counts in sputum at the end of 12-week therapy were not statistically significant, there was a correlation seen in neutrophilic elastase and neutrophil counts, highlighting their biological relevance. In Grootendorst et al. [7], the researchers mentioned that neutrophil count was significantly dropped in patients taking roflumilast. Furthermore, eosinophil, macrophages, and lymphocyte counts were also dropped in the sputum.

In comparison with the placebo, roflumilast has shown more adverse effects [17]. The adverse effects are mostly mild to moderate, but they may lead to the discontinuation of the treatment [18]. All six studies reported the adverse effects of roflumilast. These side effects were mild to moderate in intensity. The side effect associated with the use of roflumilast were upper respiratory tract infection, dizziness, weight loss, gastritis, and decreased appetite. However, Watz et al. [13] stated that if the dose gradually escalates over time, more adverse events can be avoided.

However, the current systematic review has some limitations. All studies not in the English language were excluded, and information could have been missed and limited the scope of the studies. In this review, not all the studies had open access, thus resulting in very few studies in this review.

Only one reviewer reviewed all the data, and the chance of bias was higher; it could have decreased by two or more independent viewers. The actual effect of roflumilast in COPD treatment remained unexplained as dose and duration to provide the desired effects are unclear. So, more trials are needed to be carried out for sufficient evidence to justify its role.

## Conclusions

Roflumilast is an effective drug that can be used for COPD treatment. The data included in this study are from developed countries; however, studies should be done in developing countries to study the effect on different ethnic groups. Secondly, the dose-escalation technique should be considered for further research to prevent the avoidable side effects and improve drug tolerability.
